# Supplementary material for: Efficacy and Safety of Angiotensin Receptor Blockers in a Pre-Clinical Model of Arrhythmogenic Cardiomyopathy
Source: Int J Mol Sci. 2022 Nov 11;23(22):13909. doi: 10.3390/ijms232213909 (PMC9697954; doi:10.3390/ijms232213909)
Supplement: Supplementary file 1 [file ijms-23-13909-s001.zip › ijms-2036706-supplementary.pdf]

Supplementary Figure:

Supplementary Figure S1. *Dsg2*$^{mut/mut}$ time course of disease. [3]

Supplementary Figure S2. mRNA transcription levels and echocardiography analysis of WT vehicle and *Dsg2*$^{mut/mut}$ mice treated with ARBs. (**A**) Percent left ventricular ejection fraction (%LVEF). (**B**) Percent fractional shortening (%FS). (**C**) RWT, relative wall thickness; **(D)** LVM, left ventricular mass. qPCR analysis of genes associated with **(E)** metabolism and **(F)** lipids and lipids carriers. Data presented as mean±SEM. *P<0.05 for any cohort vs WT Vehicle; †P<0.05 any Rosiglitazone-treated cohort vs vehicle-treated *Dsg2*$^{mut/mut}$ mice; #P<0.05 Rosiglitazone-treated WT vs Rosiglitazone-treated *Dsg2*$^{mut/mut}$ mice using One-way ANOVA with Tukey's post-hoc analysis.

Supplementary Tables:

Supplementary Table S1. Echocardiographic, electrocardiographic, blood pressure plethysmography, and morphometric indices from Vehicle- and Drug-treated WT and *Dsg2*$^{mut/mut}$ mice at 8 weeks of age. IVSd, interventricular septal end-diastolic volume; IVSs, interventricular septal end-systolic volume; LVIDd, left ventricular internal diameter end-diastolic volume; LVIDs, left ventricular internal diameter end-systolic volume; LVPWd, left ventricular posterior wall end diastole; LVPWs, left ventricular posterior wall end systole; FS, fractional shortening; EF, ejection fraction; HR, heart rate; RR-I, R-R interval; PR-I, P-R interval; Pd, P-wave duration; QRSd, QRS duration interval; Q-Amp, Q-wave amplitude; S-Amp, S-wave amplitude; PVC, premature ventricular contraction; BPP, blood pressure plethysmography; SBP, systolic blood pressure; DBP, diastolic blood pressure; MBP, mean blood pressure; RWT, relative wall thickness; LVM, left ventricular mass. Data presented as mean±SEM, *P<0.05 for any cohort vs WT Vehicle; †P<0.05 any drug-treated *Dsg2*$^{mut/mut}$ cohort vs vehicle-treated *Dsg2*$^{mut/mut}$ mice; &P<0.05 for *Dsg2*$^{mut/mut}$ mice treated with Valsartan vs *Dsg2*$^{mut/mut}$ mice treated with Telmisartan using Student's t-test or One-way ANOVA with Tukey's post-hoc analysis.

Supplementary Table S2: Real-time PCR run protocol and forward and reverse primer sequences.

Supplementary Figure S1

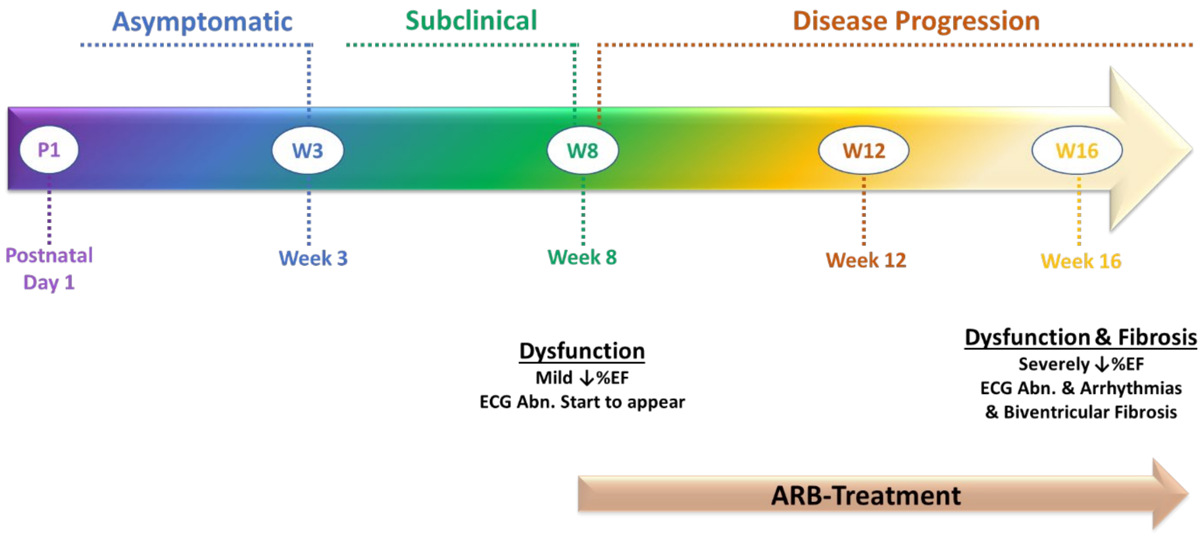

Supplementary Figure S2

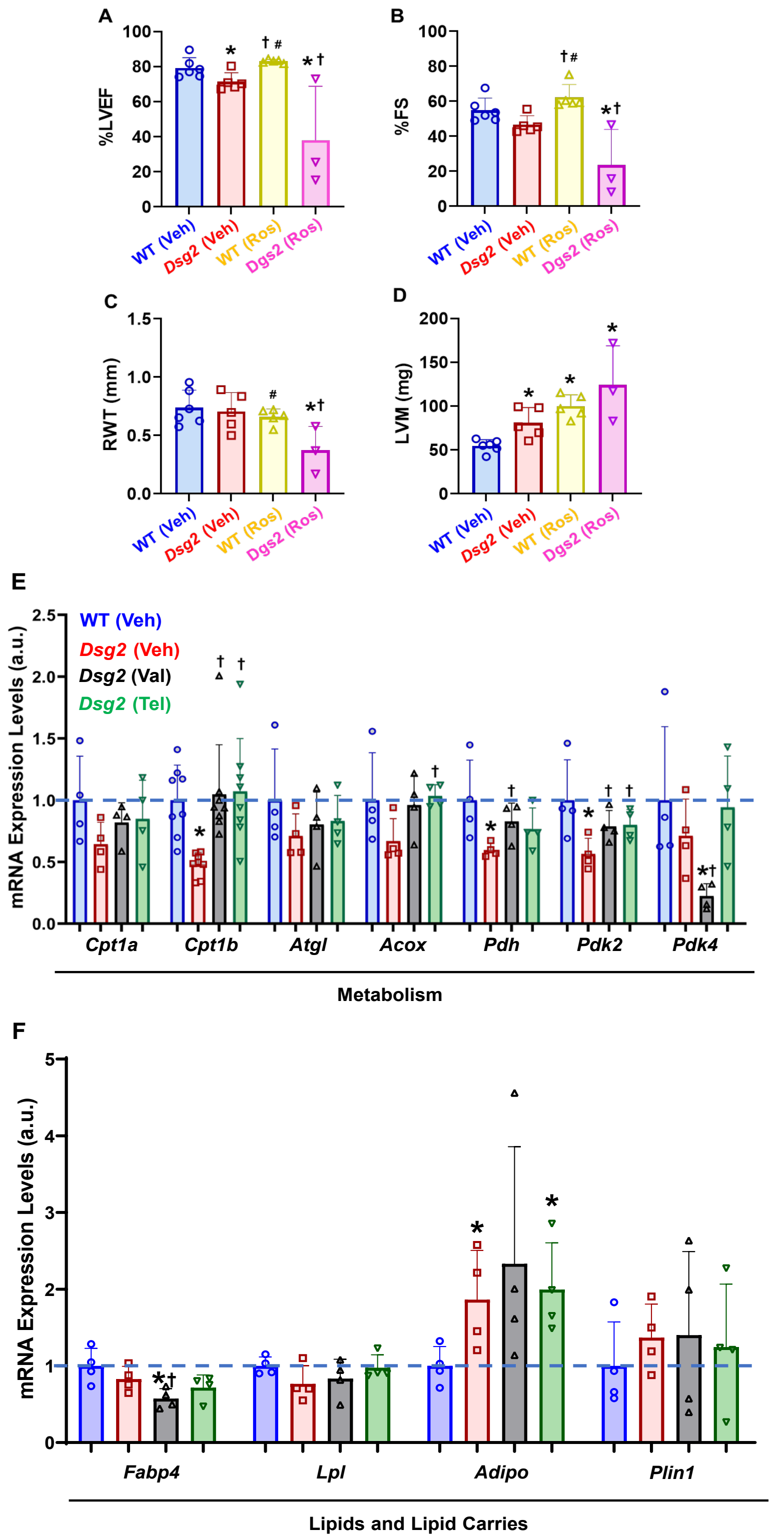

Supplementary Table S1

Parameters	Vehicle		Valsartan (30mg/kg/day)		Telmisartan (10mg/kg/day)	
	WT	*Dsg2*$^{mut/mut}$	WT	*Dsg2*$^{mut/mut}$	WT	*Dsg2*$^{mut/mut}$
**Echo**						
n	6	5	5	5	4	6
IVSd (mm)	0.85 ± 0.03	0.98 ± 0.06*	0.83 ± 0.04	0.82 ± 0.06	0.79 ± 0.15	0.79 ± 0.08*†
IVSs (mm)	1.44 ± 0.07	1.37 ± 0.11	1.28 ± 0.07*	1.21 ± 0.07*†	1.36 ± 0.12	1.21 ± 0.06
LVIDd (mm)	2.32 ± 0.12	2.66 ± 0.18*	2.15 ± 0.29	2.74 ± 0.31	2.44 ± 0.19	2.62 ± 0.23
LVIDs (mm)	1.05 ± 0.11	1.41 ± 0.05*	0.78 ± 0.14*	1.55 ± 0.31*	1.22 ± 0.19	1.24 ± 0.18
LVPWd (mm)	0.84 ± 0.05	0.92 ± 0.06	0.93 ± 0.09	0.81 ± 0.06$^{\&}$	0.81 ± 0.11	0.60 ± 0.04*†
LVPWs (mm)	0.97 ± 0.08	1.04 ± 0.04	1.20 ± 0.10*	1.06 ± 0.03$^{\&}$	0.90 ± 0.20	0.78 ± 0.10†
FS (%)	54.9 ± 3.1	46.7 ± 2.6*	64.1 ± 3.1*	44.9 ± 4.5*	50.1 ± 5.6	53.4 ± 4.0
EF (%)	79.3 ± 2.6	71.4 ± 2.6*	86.8 ± 2.1*	69.0 ± 5.4*	74.4 ± 5.3	77.7 ± 3.4
**ECG**						
HR (bpm)	433 ± 20	498 ± 14*	462 ± 6	506 ± 28*	468 ± 23	507 ± 10*
RR-I (ms)	139 ± 6.3	121 ± 3.9*	129 ± 2.0	120 ± 6.7*	129 ± 5.6	119 ± 2.4*
PR-I (ms)	36.4 ± 2.2	36.7 ± 0.5	39.3 ± 0.6	40.1 ± 1.9†	34.5 ± 3.4	36.0 ± 2.0
Pd (ms)	11.2 ± 0.8	8.3 ± 0.5*	11.5 ± 0.7	9.8 ± 0.8†	8.03 ± 0.2*	11.8 ± 1.0†
QRS-d (ms)	10.4 ± 0.6	10.3 ± 0.9	11.5 ± 0.4	12.7 ± 2.3	11.7 ± 3.3	18.3 ± 2.8*†
Q-Amp (mV)	-0.01 ± 0.01	-0.07 ± 0.05	-0.02 ± 0.0	-0.12 ± 0.06*	-0.00 ± 0.0	-0.21 ± 0.04*†
S-Amp (mV)	-0.14 ± 0.05	-0.11 ± 0.04	-0.21 ± 0.07	-0.01 ± 0.03*†	-0.16 ± 0.06	-0.04 ± 0.01*†
PVCs (%)	0.0 ± 0.0	0.34 ± 0.24	0.04 ± 0.05	0.22 ± 0.09*$^{\&}$	0.53 ± 0.22*	11.3 ± 4.2*†
**BPP**						
SBP (mmHg)	114 ± 7.7	132 ± 9.9	105 ± 2.6	114± 7.7†$^{\&}$	89.8 ± 5.9*	85.4 ± 2.5*†
DBP (mmHg)	64.6 ± 6.3	77.0 ± 10.1	58.4 ± 5.4	64.6 ± 10.2$^{\&}$	42.0 ± 1.8*	40.4 ± 1.5*†
MBP (mmHg)	81.2 ± 6.5	95.2 ± 10.0	74.0 ± 3.8	81.4 ± 9.2$^{\&}$	58.0 ± 2.4*	55.4 ± 1.0*†
**Morphometric**						
RWT (mm)	0.74 ± 0.07	0.71 ± 0.08	0.97 ± 0.26	0.62 ± 0.08$^{\&}$	0.69 ± 0.15	0.47 ± 0.04*†
LVM (mg)	54.6 ± 3.2	80.9 ± 8.7*	53.2 ± 7.3	68.0 ± 12.4	54.0 ± 7.8	49.9 ± 7.4†

Supplementary Table S2

<table>
<tr><th>mRNA gene Transcript</th><th>Forward Primer Sequence</th><th>Reverse Primer Sequence</th><th>qPCR Run Protocol</th></tr>
<tr><td>Cptb1a</td><td>GGCATAAACGCAGAGCATTCCTG</td><td>CAGTGTCCATCCTCTGAGTAGC</td><td rowspan="20"><u>Step 1:</u>
95°C for 3mins

<u>Step 2:</u>
95°C for 10secs

<u>Step 3:</u>
60°C

<u>Step 4:</u>
Go to Step 2 and repeat 60x</td></tr>
<tr><td>Cptb1b</td><td>CCAAGATCTGCTCCTACCACG</td><td>ACAGATAGCCGACGTTTGGAA</td></tr>
<tr><td>Atgl</td><td>TCCGAGAGATGTGCAAACAG</td><td>TTGGTTCAGTAGGCCATTCC</td></tr>
<tr><td>Acox</td><td>GCCATTCGATACAGTGCTGTGAG</td><td>CCCAGAAAGTGGAAGGCATAGG</td></tr>
<tr><td>Pdh-E1α1</td><td>GGTGGTGTGGTCCTAGCTGT</td><td>ATTCCTGGTGGCTGCTACAC</td></tr>
<tr><td>Pdk2</td><td>CTGGACCGCTTCTACCTCAG</td><td>GCCATCAAAGATGAGGGTGT</td></tr>
<tr><td>Pdk4</td><td>GCCTTGGGAGAAATGTGTGT</td><td>TGCTTTGATTCCTTCCATCC</td></tr>
<tr><td>PPARα</td><td>AGTTCACGCATGTGAAGGCT</td><td>AGCTCCGATCACACTTGTCG</td></tr>
<tr><td>PPARδ</td><td>GGACCAGAACACACGCTTCCTT</td><td>CCGACATTCCATGTTGAGGCTG</td></tr>
<tr><td>PPARγ</td><td>GTACTGTCGGTTTCAGAAGTGCC</td><td>ATCTCCGCCAACAGCTTCTCCT</td></tr>
<tr><td>CEBPa</td><td>AGTCGGTGGACAAGAACAGC</td><td>ACGTTGCGTTGTTTGGCTTT</td></tr>
<tr><td>CEBPb</td><td>CAAGCTGAGCGACGAGTACA</td><td>TCAGCTCCAGCACCTTGTG</td></tr>
<tr><td>PGC1α</td><td>GAATCAAGCCACTACAGACACCG</td><td>CATCCCTCTTGAGCCTTTCGTG</td></tr>
<tr><td>PGC1b</td><td>CAGCCTCAGTTCCAGAAGTCAG</td><td>CACCGAAGTGAGGTGCTTATGC</td></tr>
<tr><td>Ctnnb1</td><td>GTTCGCCTTCATTATGGACTGCC</td><td>ATAGCACCCTGTTCCCGCAAAG</td></tr>
<tr><td>Fabp4</td><td>TGAAATCACCGCAGACGACAGG</td><td>GCTTGTCACCATCTCGTTTTCTC</td></tr>
<tr><td>Lpl</td><td>GCGTAGCAGGAAGTCTGACCAA</td><td>AGCGTCATCAGGAGAAAGGCGA</td></tr>
<tr><td>Adipo</td><td>AGATGGCACTCCTGGAGAGAAG</td><td>ACATAAGCGGCTTCTCCAGGCT</td></tr>
<tr><td>Plin1</td><td>GCCACTCCAACTCCAAATGT</td><td>GCACTGCTAGGCCAGTTTTC</td></tr>
<tr><td>GAPDH</td><td>CATCACTGCCACCCAGAAGACTG</td><td>ATGCCAGTGAGCTTCCCGTTCAG</td></tr>
</table>
